# On the Various Modes of Treating Intermittents in Paris

**Published:** 1830-07-01

**Authors:** 


MODES OF TREATING INTERMITTENT FEVER PURSUED AT THE VARIOUS HOSPITALS OF PARIS.*

It will be curious, and may be useful, to notice a resumé of the Parisian modes of treating intermitting fever, which has lately been published in a French contemporary. It is singular that scarcely two physicians in this metropolis treat an ague in precisely the same manner, though all agree in the principle of administering bark in one of its many forms. One will commence with a vomit; another with a purge; a third will neither vomit nor purge but proceed at once to the cinchona; and a fourth, whom we take to be the most judicious of the whole, will adapt his emetic, or his calomel and jalap, or his sulphate of quinine to the duration of the complaint, the character of the accompanying symptoms or lesions, and the relative condition of the patient. Let us see how matters stand with our Parisian confrères.

It appears that in Paris, as in this country, intermittent fevers have been more prevalent within these last few years, than they had been for some time previously. The ratio principii, or cause, is keenly disputed on the other side of the water, and some local circumstances are thought by one party to afford an explanation of the circumstance. This may, in part, be true, but the general occurrence of aguish complaints in various parts of Europe which had latterly been free from their visitations, must depend on some more potent and extensive influences. It is more than probable that these exist in the atmosphere rather than the earth, for the seasons have exhibited considerable alterations from the ordinary and even " tenour of their way," since 1825.

HÔPITAL BEAUJON.

During the year 1827, one hundred and eighteen patients affected with intermittent fever have been admitted into this hospital; their ordinary time of remaining in the institution is thirteen days. Of these 118 patients 96 were males, and 22 females, but as the beds for the former are one sixth more numerous than those for the latter, the calculation will be 82 men to 20 women, or as 4 to 1. No doubt the causes of this great disparity between the liability of the sexes to ague, must be looked for in their different habits of life, as well as in the circumstances of profession and exposure. Forty-two of the individuals were above thirty years of age, seventy-six below it. Twelve cases occurred in Winter, thirty-seven in Spring, forty-two in Summer, and twenty-seven in Autumn. The quartans predominated in Winter, the tertians in Spring and Autumn, and the quotidians in the Summer. The majority of the patients from the country were from Boulogne, Boint-du-Jour, or other such damp localities, whilst the Parisians were mostly inhabitants of the dark narrow streets in the vicinity of the Seine, or persons with sedentary and unwholesome occupations.

The writer of the foreign article on which we are now employed, who appears to be an offset from the " physiological" trunk in the Val-de-Grace, in other words a disciple of Broussais, lays down ths following rules of treatment, founded on those which guide that celebrated systematist. 1mo. The first means should be directed against the irritation in the system, the removal of which generally removes the fever also : our author has seen *many* of these affections, intermittents, yield to antiphlogistics only at the Val-de-Grace ;* 2ndo. If symptoms of gastric or intes-

---

* Journ. Complément. Février, 1830. No. 140.

* The Broussaians are then more fortunate than we. Is it because their doctrine teaches that they *should* be so ?

tinal phlegmasia be present we should abstain from administering febrifuges in the first instance; 3tio. When the irritation is confined to the mucous membrane of the primæ viæ,* it is proper to administer them by the colon, and vice versâ; 4to. The febrifuges, especially quinine and its preparations, being viewed as irritants, should only be employed in small doses. Making some allowances for modes of expression and national usages, as in the *lavement* proposition, the above rules are very good ones and deserving of more consideration than they often seem to receive in practice.

In the 118 cases that occurred at the Hospital during 1827, M. Renauldin, the physician in charge, pursued the following method with universal success. After the first paroxysm six grains of the sulphate of quinine in three pills were given until two periods had passed over without a fit; the same medicine was then continued for eight days, the dose being gradually diminished, and a pill being given from hour to hour in such a manner that the last was taken two hours before the expected paroxysm. The diet was good until the cessation of the fever.

### HÔTEL DIEU.

M. Husson, one of the physicians to this establishment, gives the sulphate of quinine internally unless there be evident counter-indications. He begins with doses of one or two grains, which he augments progressively and indefinitely, according to the obstinacy of the complaint. A severe tertian was arrested in a girl of sixteen by a single grain dose of the sulphate.

M. Recamier usually begins with four or six grains of the quinine, and increases the dose daily, if necessary, to twelve, fifteen, or eighteen grains in the twenty-four hours. Such is the treatment of *ordinary* cases by the other physicians of the Hôtel Dieu, as well as by those of the Charité and other institutions of Paris.

M. Bally, who believes in the *essentiality* of fevers, in the most ancient and extended sense of the term, maintains that the sulphate of quinine is only an irritant when given in small and repeated doses. Accordingly he prescribes it in very large ones, beginning with thirty-six, forty, or even sixty grains in the twenty-four hours. M. Bally asserts, that this practice not only arrests fevers promptly but prevents the occurrence of the organic alterations that are too often left behind. Like those who pursue the very opposite plan, M. Bally can appeal to a number of successful cases. This physician has been recently experimenting on the *salicine*, or principle obtained from the bark of the willow. In the case of a young pregnant woman, who attributed her complaint to terror, and suffered from *two fits* during the day, the fever was allowed to run on for seven days, and eighteen grains of the salicine in three doses were then prescribed. The remedy was continued for the two succeeding days, when its use was discontinued on account of some irritation which it seemed to produce in the throat; the fever was perfectly arrested. The reporter adds that several other equally conclusive cases have occurred in favour of this medicine.

The ligature of the limbs has been tried several times at the Hôtel Dieu, and with occasional success, but not sufficient to inspire any extraordinary opinion of its powers in the minds of the experimenters.

### LA CHARITÉ.

Experiments have been made at this hospital on the febrifuge powers of the misletoe in powder, which has lately been represented as more efficient than even the sulphate of quinine. M. Chomel has employed it on five or six patients during the course of the last Autumn, but without success. The following facts deserve to be recorded and remembered. It is not because the virtues of a miserable drug like the misletoe, if drug it can be called, are put in question, but because the same

* By primæ viæ, the *upper* portions of the intestinal tube are obviously alluded to.

circumstances step in to disturb our reasonings and vitiate our conclusions with respect to more potent and efficacious articles of the materia medica. The fact then to which we would draw the attention of our readers is this—M. Chomel being desirous of testing the powers of the misletoe, selected, last Autumn, *twenty-two* patients labouring under intermittent fever. Before exhibiting the medicine he waited for the appearance of some paroxysms, and the consequence was, that in *seven* the fever ceased spontaneously, and a cure ensued without the aid of any medicinal remedy whatever. In *four* other patients the paroxysms gradually and spontaneously diminished, and required a very small dose of the quinine for their complete dispersion. Of the eleven remaining individuals, *eight* displayed symptoms of intermittent phlegmasia, and were cured by antiphlogistics; and the final three, who alone became subjects for the misletoe, experienced no benefit from its use but were cured by the quinine. This does not prove much in favour of the misletoe.

Here we must conclude, and perhaps we may be allowed to observe that so long as good bark is to be procured, practitioners will trust little to the inferior remedies which chance or ingenuity may point out as its substitutes. If a time shall arrive when cinchona is no more, or so scarce as to be sealed to all but the gold of the wealthy, then, and not before, will the numerous indigenous or foreign bitters be put into requisition for the treatment of ague. At present, bark and arsenic are worth ten times more than the whole of them.

